# Rearfoot strikers have smaller resultant tibial accelerations at foot contact than non-rearfoot strikers

**DOI:** 10.1186/1757-1146-7-S1-A93

**Published:** 2014-04-08

**Authors:** Molly D Glauberman, Peter R Cavanagh

**Affiliations:** 1 Deparment of Orthopaedics and Sports Medicine, University of Washington, Seattle, WA, 98195, USA

## Purpose

Overuse injuries are common in recreational runners. Recent reports have implicated the characteristics of the footstrike in the etiology of stress responses in the tibia. This has motivated efforts to modify the loading at footstrike by altering the orientation of the foot at first contact. The present study aimed to: 1) report typical magnitudes of resultant tibial acceleration (TA) in women distance runners; 2) contrast TA in rearfoot and non-rearfoot striking runners; and 3) examine TA during non-natural footstrike patterns in runners.

## Method

We used a leg-mounted tri-axial acceleration monitoring unit to measure TA and angular velocities. Twenty injury-free women distance runners (age 27.8±3.7 years, height 168.1±6.2 cm, body mass 59.2±7.3 kg, weekly mileage >20) participated in the study. The sensor was positioned 5cm above the medial malleolus along the medial tibial border and tensioned to 22N with a Velcro strap. Multiple 60-second running trials at 3.13 m/s on a force-measuring treadmill (Kistler 9287 plate) were collected.

## Results

**Figure 1:**
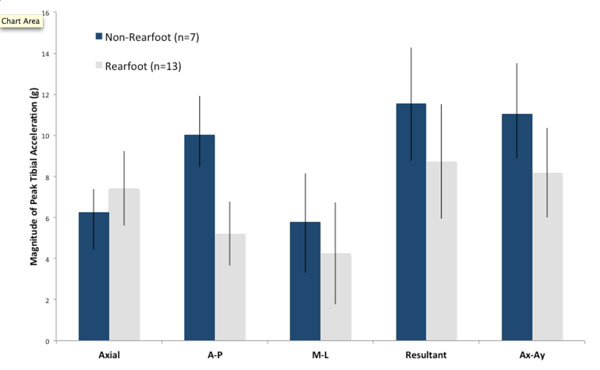
Peak tibial acceleration in rearfoot (n=13) and non-rearfoot (n=7) strikers in Brooks Adrenaline shoes.

**Figure 2:**
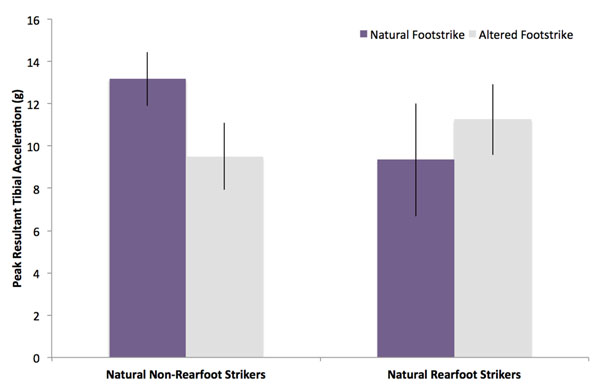
Peak resultant tibial acceleration in runners who ran with an altered footstrike (n=18) while wearing their own shoes.

The range of values for axial peak tibial acceleration (PTA) in the group was 4.6g to 10.9g. Axial PTA in 7 non-rearfoot strikers (6.3±1.1g) was not significantly different from that in rearfoot strikers (7.4± 0.8g; p=0.15). However, the anterior-posterior acceleration component and the resultant PTA in non-rearfoot strikers (10.0±1.9g) were significantly greater than that in rearfoot strikers (5.2±1.6g; p=<0.001) (Figure 1). In a second part of the study, twelve natural rearfoot runners were instructed to change their strike pattern to a non-rearfoot strike while 6 non-rearfoot strikers changed to a rearfoot pattern. The average resultant PTA for the natural rearfoot strikers increased from 9.4g to 11.3g, whereas 6 natural non-rearfoot strikers decreased to 9.5g from 13.2g when switching to rearfoot striking (Figure 2).

## Conclusions

It is not advantageous for rearfoot strikers to transition to non-rearfoot striking if PTA is the criterion measure. Previous studies that have only examined the axial component of tibial acceleration may have reached the wrong conclusion because the A-P component is the larger component in non-rearfoot strikers. Our findings suggest that a transition away from rearfoot striking is likely to increase tibial acceleration at footstrike. Thus, if tibial stress injuries are indeed related to resultant tibial acceleration at footstrike, a change to non-rearfoot striking may increase the risk of injury.

